# Spiking Neurons Integrating Visual Stimuli Orientation and Direction Selectivity in a Robotic Context

**DOI:** 10.3389/fnbot.2018.00075

**Published:** 2018-11-20

**Authors:** André Cyr, Frédéric Thériault, Matthew Ross, Nareg Berberian, Sylvain Chartier

**Affiliations:** ^1^Conec Laboratory, School of Psychology, Ottawa University, Ottawa, ON, Canada; ^2^Department of Computer Science, Cégep du Vieux Montréal, Montreal, QC, Canada

**Keywords:** spiking neurons, vision, direction selectivity, orientation selectivity, motion detection, artificial intelligence, robot

## Abstract

Visual motion detection is essential for the survival of many species. The phenomenon includes several spatial properties, not fully understood at the level of a neural circuit. This paper proposes a computational model of a visual motion detector that integrates direction and orientation selectivity features. A recent experiment in the Drosophila model highlights that stimulus orientation influences the neural response of direction cells. However, this interaction and the significance at the behavioral level are currently unknown. As such, another objective of this article is to study the effect of merging these two visual processes when contextualized in a neuro-robotic model and an operant conditioning procedure. In this work, the learning task was solved using an artificial spiking neural network, acting as the brain controller for virtual and physical robots, showing a behavior modulation from the integration of both visual processes.

## 1. Introduction

Visual motion detection (MD), direction selectivity (DS) and orientation selectivity (OS) are essential basic mechanisms for processing visual input from the environment (Borst and Euler, 2011; Clark and Demb, 2016; Nath and Schwartz, 2016). However, the neural correlates at the level of cellular circuitry are not fully understood (Takemura et al., 2013). The study of elementary MD and DS models under the umbrella of computational vision is based on a few theories (Hassenstein and Reichardt, 1956; Hubel and Wiesel, 1959; Barlow and Levick, 1965). The basic algorithm of a MD relies on the integration across space and time of a moving light or dark stimuli (Yonehara and Roska, 2013; Behnia et al., 2014), while DS property is mainly achieved from facilitating the response to preferred motion and/or inhibiting the response to the null motion (Clifford and Ibbotson, 2002; Fried et al., 2002; Li et al., 2014; Mauss et al., 2015; Salay and Huberman, 2015).

Several studies have used the well-known Drosophila model in vision science (Paulk et al., 2013), validating underlying mechanisms of DS (Eichner et al., 2011; Gilbert, 2013; Maisak et al., 2013; Shinomiya et al., 2014; Leong et al., 2016; Haag et al., 2017). Recently, insights from the Drosophila brain have shown that few visual neurons display both directional tuning and orientation selectivity (Fisher et al., 2015). Notably, when the axis of motion is orthogonal to the orientation of the moving stimulus, directional tuning is sharpened. As the orientation of the moving stimulus aligns in parallel to the direction of the axis of motion, neuronal responses are reduced.

Orientation and direction selectivity from retina to cortex were experimentally shown in various mammalian species and rigorously quantified from statistics methods (Borg-Graham, 2001; Mazurek et al., 2014). The emergence of DS and the influence of visual experience are extensively studied (Li et al., 2008; Haag et al., 2016; Leong et al., 2016; Strother et al., 2017) as well as the elaboration of computational models (Mu and Poo, 2006; Elstrott and Feller, 2009; Berberian et al., 2017). One possibility may consist of a bias architecture early in the neural development toward specific preferences (Adams and Harris, 2015). There is also computational work suggesting that spontaneous activity appearing during the early stages may give rise to the emergence selectivity features (Van Hooser et al., 2012). The foundation of this may find echoes in the genetics and from the primary units in the retina that already compute and provide the information at that level.

This research presents a spiking neural network (SNN) model to study the interaction between visual orientation and direction selectivity features in a MD model that responds to basic visual motion stimuli. SNN is a relevant computational method to use given the temporal property that helps capture dynamic and coincidental events (Maass, 1997) using spike-timing-dependent plasticity (STDP) (Bi and Poo, 1998; Feldman, 2012). However, SNN remains poorly used regarding the MD and DS visual topics (Shon et al., 2004; Wenisch et al., 2005) especially in complete embodied models. A key advantage of using physical robots in neural modeling is to validate models under real world constraints (Webb, 2000).

Bio-inspired neural models in vision (Kerr et al., 2015) and motion detector models (Franceschini et al., 1992) are not new topic in neuro-robotics. Simulation of a stabilization and fixation robotic behavior from a motion stimulus reproduce mainly the visuomotor process of the fly (Huber and Bülthoff, 1998). But merging OS and DS in SNN paradigm is still unexplored. Furthermore, given that the relationship between orientation and direction selectivity remains to be investigated at the behavioral level, this research aims to embody these two related processes in virtual and physical robots as a proof of concept (Pezzulo et al., 2011; Krichmar, 2018). In this perspective, the present model was evaluated under an operant conditioning context, modulating its behavioral response when shown basic orientated stimuli in motion. More precisely, a detailed framework to trace dynamical visual stimuli from sensors to motors is proposed, which could be used in future robotic implementations in the computational vision domain.

In this experiment, the operant conditioning learning process (Cyr et al., 2014) is used as behavioral context. As such, a reward mechanism reinforces connections amongst units coding for the preferred direction of motion in relation to its neutral actions. The application of a positive reward provides the advantage of starting off with no initial assumption about the underlying behavior of units exposed to stimuli in motion. From this learning procedure and with the knowledge of the orthogonal (orientation/motion) aspect of a stimulus, a fasten motor response is proposed. The contribution of this paper is to introduce a bio-inspired model of motion detector integrating direction and orientation selectivity features, implementing these processes at a behavioral robotic level.

The next sections detail the SNN architecture and the simulation environments used, followed by an analysis of the obtained results. It concludes with a discussion on the model and its future perspectives.

## 2. Methodology

The goal is to simulate an enhanced behavioral response of a virtual and physical robot, when the orientation of a visual stimulus is orthogonal to its motion. From an operant conditioning procedure, the robot learns to link a positive reward with actions of lighting up LEDs and choosing the desired solution. The additive effect of the orientation and motion features of a visual stimulus was demonstrated in the Drosophila, a challenge to model in a neurorobotic paradigm.

### 2.1. Protocol

**Figure 1:**
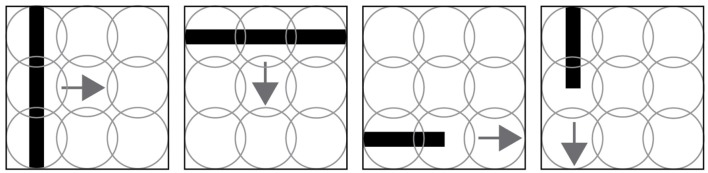
Four trial examples made in the experiment. The first two images show a line moving orthogonal to its orientation. The last two images show lines moving in their same orientation. Those four scenarios are evaluated for both directions, for example a horizontal line moving from left to right and right to left. The circles, representing the sensory receptors of the visual field, overlap to reduce unseen areas.

**Figure 2:**
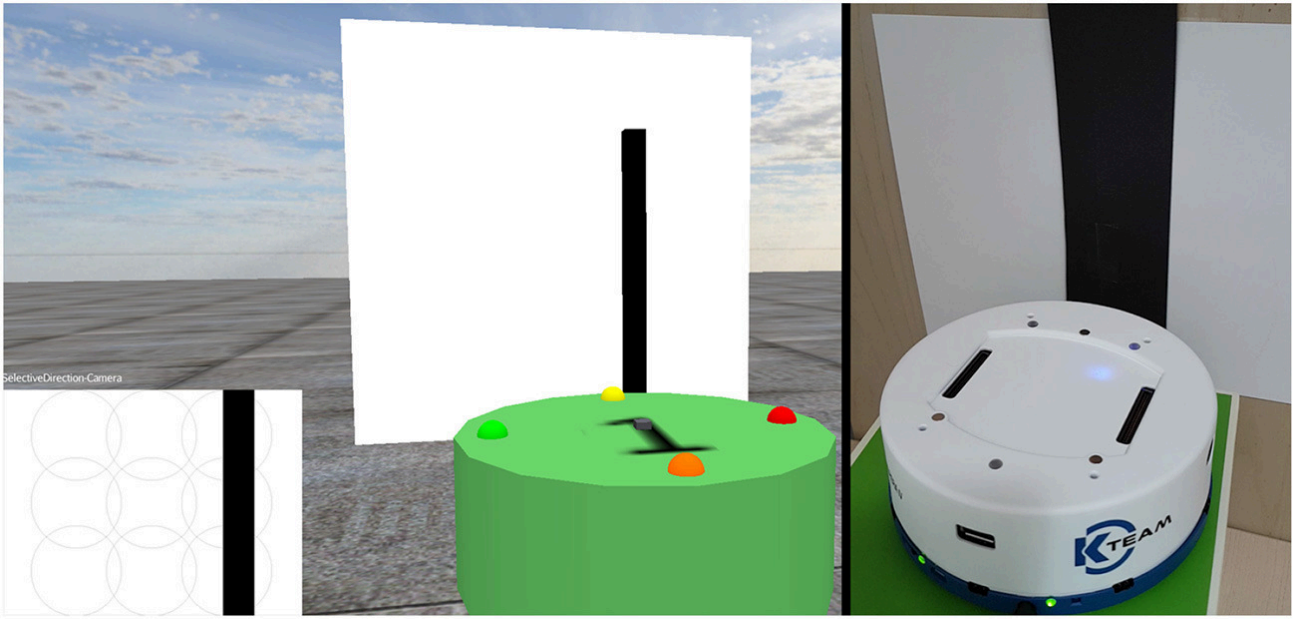
Virtual and real world environments, showing the robots in front of an image. The bottom left section represents what the virtual robot currently sees with its camera.

The virtual experimentation consists in displaying black lines (horizontal and vertical) that move horizontally and vertically in front of a robot (see Figures 1, 2). The SNN architecture, as well as the 3D world experiment were elaborated with the SIMCOG software (Cyr et al., 2009). Four scenarios were evaluated in the two opposite motion directions, for a total of 8 different trials. In each of them, a line passes from one end of the retina to the other. In this study, the retina is composed of a 3 × 3 sensory neurons matrix.

Each combination of orientation/direction trial is repeated several times in order to reach the learning criteria. The sequence of trials is pre-determined. At the beginning of the simulation, the robot randomly chooses an action, by blinking a light on one of its four possible LEDs, following the capture of a moving visual stimulus. The robot learns from a supervised positive reinforcement to correctly associate the desired output with its own previous actions. Finally, the simulation ends with the evaluation of the behavioral response combined with the orthogonal/non-orthogonal feature of stimuli (motion/direction). In this study, foreign patterns were not tested, since the other stimulus features were not used (only vertical/horizontal and motion).

The virtual simulation lasted 24,000 cycles (3,000 cycles for each trial). A particularity of the software is that it works using cycles instead of milliseconds. This allows computers of various power to have the same output at the end. The approximation conversion is 10 ms/cycle for the virtual experiment, running on an i7 desktop computer.

### 2.2. Architecture

**Figure 3:**
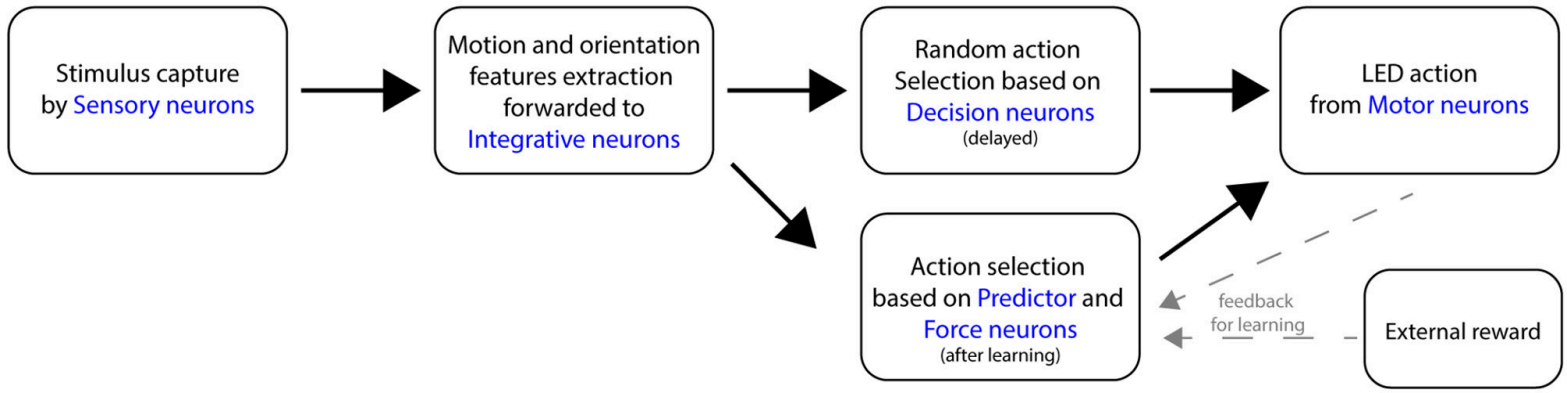
Flow diagram of the main components of the SNN, following the perception of a moving stimulus in front of the robot.

The general topology of the SNN consists of several neural layers, as shown in Figure 3. A sensory layer captures the visual stimuli, then the orientation and motion features are extracted and forwarded to an integrative neural layer. A Decision layer then proposes a random action to the motor layer. Once the learning is completed, A Force neural layer overrides the initial random decisions. Following a desired output, an external positive reinforcement is applied to the robot and caught from arbitrary dedicated Reward neuron.

**Figure 4:**
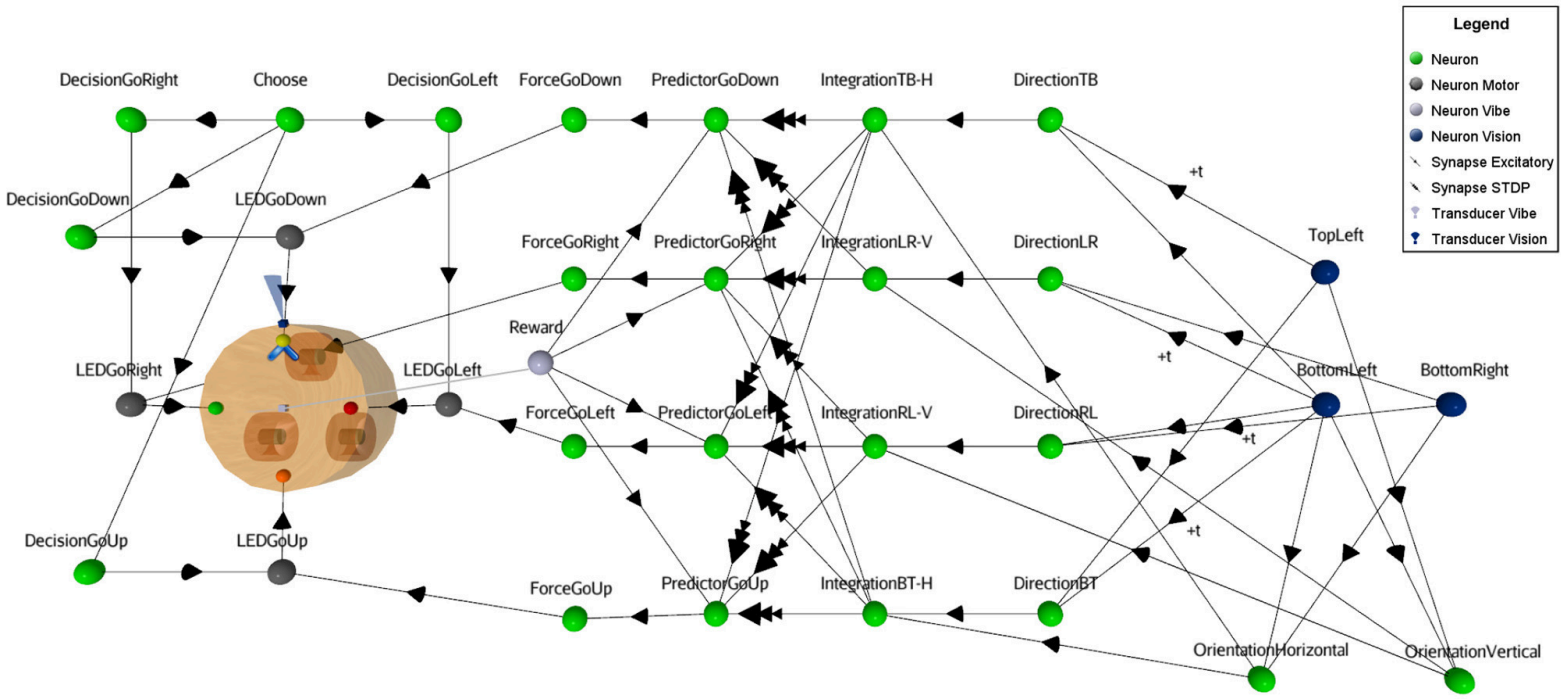
Simplified SNN architecture. Following the reception of a moving visual stimulus perceived from Vision neurons, the linked Direction and Orientation neurons forward the information to Integration neurons. At this point, the features of the stimulus are obtained and used for the learning task. This involves Predictor and the Reward neurons to enhance the synaptic links from the STDP function (i.e., synapses between Integration and Predictor neurons). In the virtual environment, vibrations act as rewards, hence the gray square at the center of the robot that represents the vibration transducer. When the rule is learnt, Force neurons bypass the random Decision neurons to trigger the appropriate action (i.e., LED action neurons).

For illustrative reasons, a simplified neural circuit based on three sensory neurons instead of the full 3x3 matrix is shown in Figure 4 (see complete SNN architecture and the table values of neural parameters in Supplementary Material at http://aifuture.com/res/2018-dir). Though it is sufficient to highlight the visual mechanism of an enhanced motion/orientation neural response.

**Figure 5:**
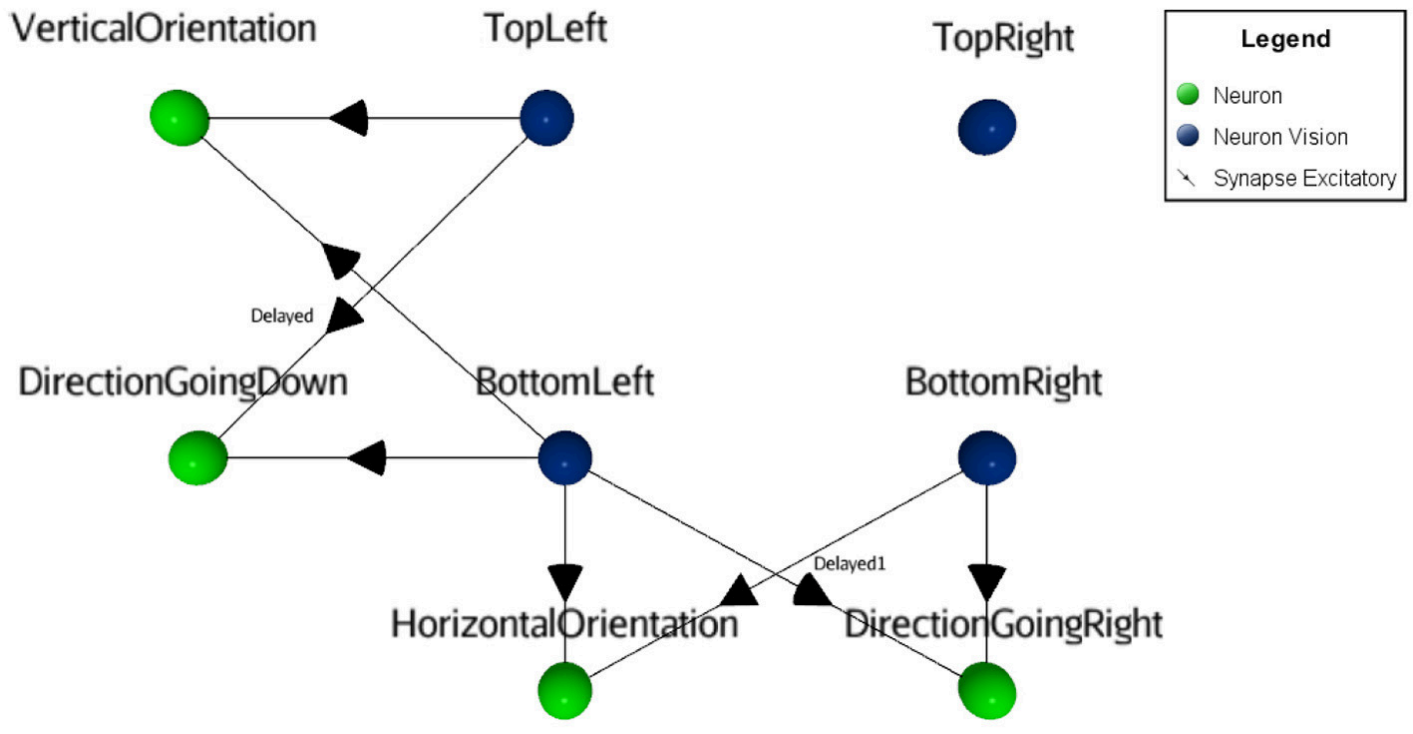
Visual orientation and direction selectivity features extraction process. The orientation as well as the motion information (green neurons in this figure) are obtained by merging two adjacent sensory neurons (blue neurons). For the motion feature, delayed synapses were used.

On the left part of Figure 4, one can see that the robot has four different binary output responses. These consist of LEDs located at the four cardinal points on top of the robot. Each are attached to their respective motor-neurons (LEDGoRight, LEDGoDown, LEDGoLeft,LEDGoUp). This visual computational model includes a camera at the front of the robot (next to the yellow LED on the figure) and a sensor to capture the reward. In this study, a vibration sensor was chosen and is automatically triggered by the virtual environment when a proper decision is made. The visual black bars stimuli are caught by the camera and their linked sensory neurons (blue circles), see right part of the figure. At this point, Sensory neurons fall under a cooldown period using a refractory period parameter, to prevent the constant capture of stimuli. Then, these neurons forward the signal to both motion and orientation neurons. In case of motion, the neurons receive inputs with temporal fixed synaptic delays (see Figure 5) to achieve the integration.

For simplicity, instead of introducing inhibition in the neuronal responses for motion in the non-preferred direction as in the natural model, this SNN uses identical dedicated excitatory synapses for both opposite directions. Thus, direction and orientation neurons connect with excitatory synapses on integration neurons, providing the orthogonal feature (motion with the inverse orientation). Those integrative neurons are linked to Predictor neurons with a weak synaptic link. Therefore, the Integrative neurons cannot trigger Predictor neurons alone. Predictor neurons also receive a positive reinforcement signal from a Reward neuron (vibration sensory neuron), in order to meet the pre/post timing criteria relation of the learning rule. When a Predictor neuron spikes, it forwards signal to the Force neuron that will trigger the output response. Since there are multiple synapses between the Integrative neurons and the Predictor neurons, the behavioral plasticity is summed between the motion neurons and the actions of the robot. Finally, Motor neurons (gray circles in Figure 4) output to the attached LEDs. The result of the learning process is that after a certain time, rewards will not be necessary anymore to forward the signal through the circuit. Once an action is selected, all other actions are inhibited from a dedicated neuron (not shown in Figure 4, for visibility).

### 2.3. Neural dynamic

The spiking neuron model includes standard features such as a time varying membrane potential variation, a refractory period, a threshold and a spike emission state (Equations 1, 2, 3, and 4). These neurons are connected through dynamical excitatory and inhibitory synapses, and some are modulated from a spike-timing-dependent plasticity (STDP) learning rule (Equation 5). Equation (2) represents a nonlinear potential variation simulating an excitatory or inhibitory postsynaptic potential (PSP). This architecture also uses some fixed PSPs with different lengths to regulate control neural inputs.

In this study, the learning rule from STDP needs a third factor (the reward) to be activated (Frémaux et al., 2010; Kuśmierz et al., 2017) (STDP-R). A negative reinforcement (punishment) could have been used to modulate the learning curve, but it was not implemented. Mainly, the objective is to reach a specific synaptic weight value in order to force the proper action upon an associative event. This value depends on the initial synaptic weight and the increase step of the STDP function (100%, as specified in Equation 5). Also, to prevent overshooting this threshold, a capping value was specified to 300%.

**Equation 1: Leaky integrator neural dynamic**.

$$v_{m}\left(k\right)=f\left(v_{m}\left(k-1\right)+\sum v_{i}\right)$$

**Table:** 

*v*_*m*_(*k*)	= membrane potential at cycle k
*v*_*i*_	= synaptic input as calculated in equation 2
*f*	= membrane potential curve as calculated in equation 3

**Equation 2: General function describing the postsynaptic potential curve**.

$$v_{i}\left(t\right)=\left\{ \begin{array}{ll} ae^{-t/\tau }\; & ift\leq tMax\\ 0\; & ift>tMax \end{array}\right.$$

**Table:** 

*a*	= maximum amplitude (set to 20)
τ	= tau (i.e. 8)
*t*	= time since spike (in cycles)
*tMax*	= maximum duration of a PSP (set to 15 cycles)

**Equation 3: Membrane potential function**

$$f\left(v_{m}\right)=\left\{ \begin{array}{ll} g\left(v_{m},0\right)\; & ifv_{m}<v_{m}Rest\\ v_{m}Rest\; & elseifv_{m}=v_{m}Rest\\ g\left(v_{m},1\right)\; & elseifv_{m}<v_{m}Threshold\\ 100\; & else \end{array}\right.$$

**Table:** 

*g*(*v*_*m*_, 0)	= see equation 4
*v*_*m*_*Rest*	= membrane potential rest value (set as 43)
*v*_*m*_*Threshold*	= threshold value (set as 65)

**Equation 4: Membrane potential output**

$$g\left(v_{m},d\right)=\left\{ \begin{array}{ll} min\left(eachvinvecwherev>v_{m}\right)\; & ifd=0\\ max\left(eachvinvecwherev<v_{m}\right)\; & ifd=1 \end{array}\right.$$

**Table:** 

*vec*	= [4, 11, 18, 23, 28, 32, 36, 42, 43, 44, 45, 47, 50, 53, 58, 65, 100]
	Ascending phase to reach threshold = *exp*(0.8+0.3*t)+40*foreachtfrom*0*to*8
	Ascending phase from post action potential to rest = *log*10(0.9+0.2*t)*100*foreachtfrom*1*to*7
	Action potential = 100

**Equation 5: General STDP function**.

$$\Delta w=b*\alpha_{t_{post}-t_{pre}}e^{\frac{t_{post}-t_{pre}}{\pi }}$$

**Table:** 

Δ*w*	= synaptic weight change
α_*t*_*post*_−*t*_*pre*__	= 1 or −1, depending on the sign of *t*_*post*_−*t*_*pre*_
π	= time constant
*b*	= bias factor (1.0 for + timing, 1.0 for - timing)

STDP coefficients for Δ *w*:

Effect duration = 24,000 cycles

Max. synaptic change in one paired spike = 100%

Max. synaptic change = 300%

Max. STDP time window = 100 cycles

### 2.4. Physical environment

A physical simulation was done to better evaluate the ability of the SNN to operate under suboptimal timings and conditions. In this environment, the SNN model was embedded in a Khepera IV robot (https://www.k-team.com/khepera-iv), with two modifications. First, instead of using a vibration sensor, the reward was given to the robot using the back infrared. Also, since the robot contains only three programmable LEDs, different colors were used to explicitly referred the four possible directions. These minor changes do not affect the functional aspect of the SNN.

## 3. Results

**Figure 6:**
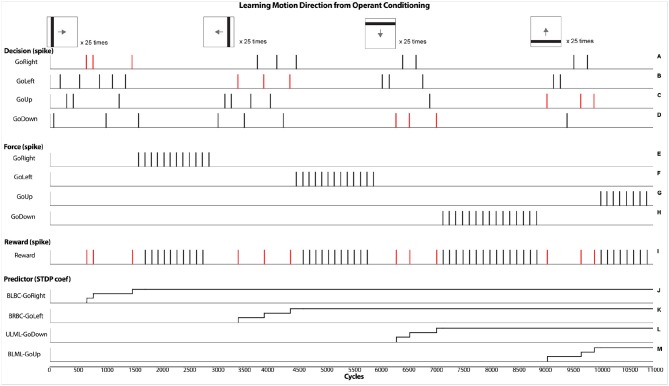
Results obtained from the virtual experiments. It represents the operant conditioning learning process that associates a motion direction of a visual stimulus with an external supervised reward. Specifically, after a randomized decision (graphics A,C,E,G), if a reward is given (graphic I), the associated predictor is allowed to spike, followed by a forced action (graphics B,D,F,H) from the STDP learning rule (graphics J–M).

Figure 6 shows the dynamic of few main neural components that reflects the learning process of motion direction from an operant conditioning procedure. At the top, small images represent the displayed lines including their orientation and direction. Each of them is repeated several times. At the beginning, when the robot detects a visual stimulus motion, it randomly activates a LED. This output is represented in graphics A, C, E, G from triggering one of the four possible Decision neurons (GoDown, GoUp, GoLeft, GoRight). If the decision corresponds to the good motion direction, then an external supervised reward (graphic I) is sent to the robot. This reinforces the associated Predictor neuron. The pairing of the pre/post spikes and the STDP learning process (graphics J–M) results in an increase of synaptic weights along the operant conditioning procedure. In this simulation, STDP parameters are tuned to trigger Force neurons (Graphics B, D, F, H) with only three correct associations. In the graphics J–M, three steps are shown indicating the learning process. Learning curves are determined from two factors, the preset synaptic weight and the learning incremental step. This rapid learning was done to reduce the number of trials in the experiment considering the four possible directions as well as four possible output responses. The result of the learned association consists in overriding the random decision with Force neurons to trigger the proper action.

**Figure 7:**
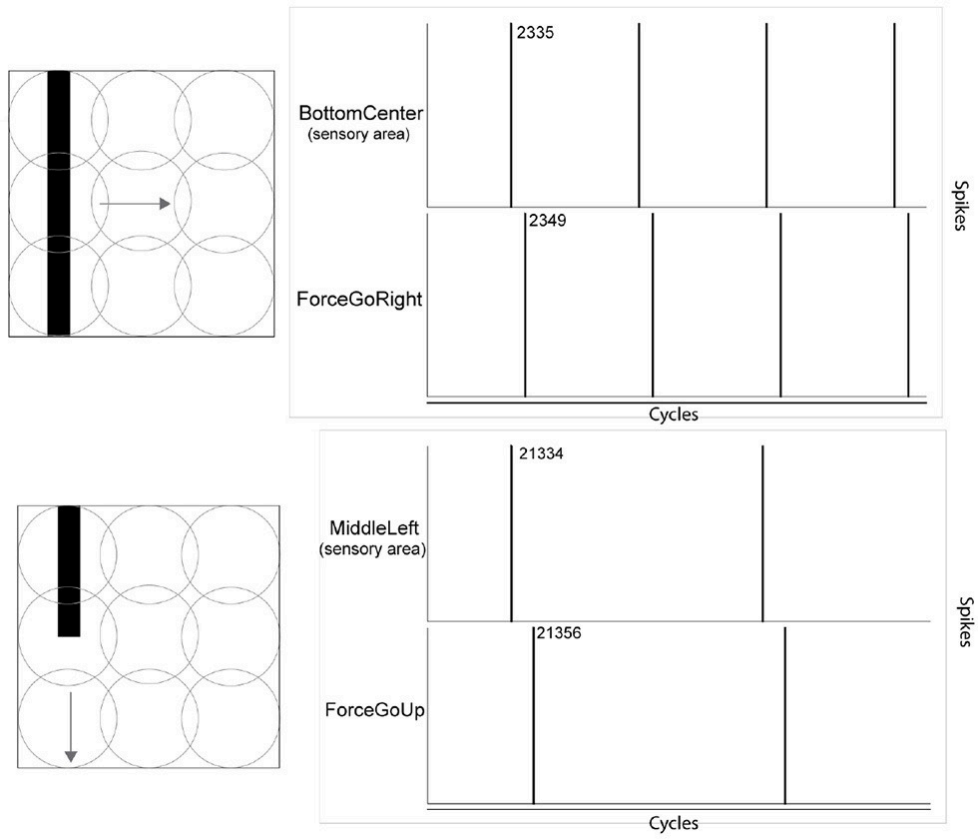
Effect of merging the orientation feature of a visual stimulus and its motion direction feature. The onset of the motor response arrives earlier when the orientation is orthogonal to its motion. Values in the figure refer to algorithm cycles.

In the experiments, the effect of orthogonal orientation of a visual stimulus in relation to its motion can be observed. In Figure 7, two scenarios are shown: a vertical bar moving horizontally, and a vertical bar moving vertically. In the upper right part, the graphic highlights the detection of the stimulus at a precise moment in the experiment and the Force GoRight neuron spikes accordingly after the learning process. The absolute timing difference is 14 cycles in this orthogonal orientation/motion trial. In the bottom part of the figure, the graphic caught the vertical stimulus moving in the vertical axis from precise spiking and the Force GoUp trigger also spikes after but with a longer period of onset with 23 cycles of difference. This motor response gained is identical in the opposite scenario, a horizontal bar moving vertically giving faster onset response than a horizontal bar moving horizontally.

**Figure 8:**
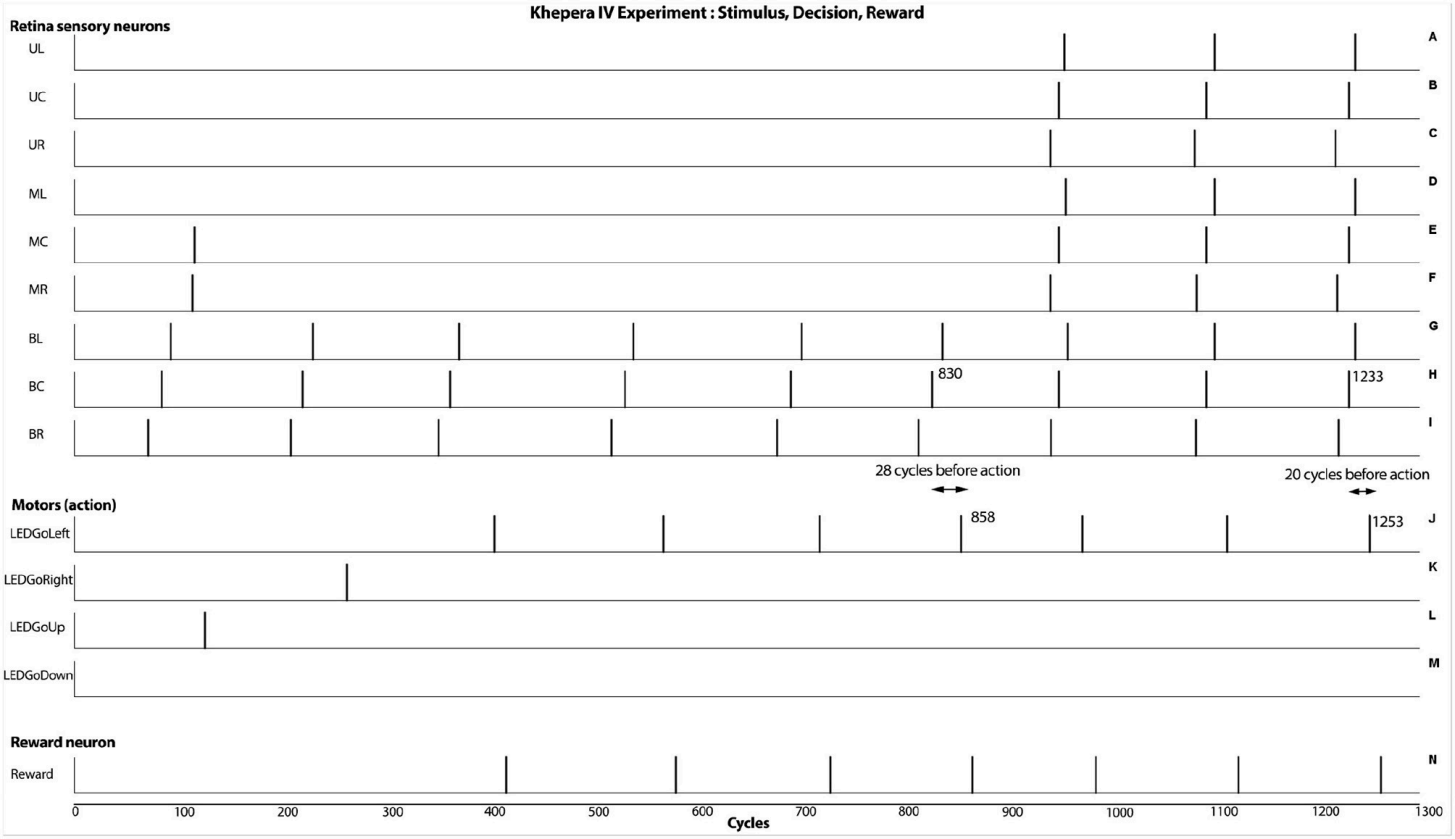
Results from the real experiment (Khepera IV robot). As seen at cycles 858 and 1,253, the robot is able to react after the capture of the stimulus. This result shows an additive behavioral response from integrating the motion and the orthogonal orientation of the visual stimulus. In this case, a fasten responses of approximately 30% can be obtained.

Under the Khepera IV robot, similar results were obtained, even with less precise timing of events. In Figure 8, graphics H and J show that the absolute timing difference between detecting the movement and opening a LED is around 28 cycles when an horizontal line was moving horizontally. When showing a vertical line moving horizontally, the timing difference is reduced to 20 cycles, hence having a response time 30% faster. This behavioral change and its concrete outcome is dependant of the robotic scenario.

## 4. Discussion

The proposed SNN architecture sustains basic visual orientation and direction selective processes. Integration of these two stimuli features in dedicated neurons was shown in the Drosophila model, sharpening the direction neuronal responses (Fisher et al., 2015). Moreover, a preference association was found when the orientation of the stimulus was orthogonal to its motion. These phenomena were successfully simulated in the proposed SNN model using a precise design of synaptic connections to reproduce the functional outcome at a robotic behavioral level. This, in the neural-robotic domain, suggests that merging two or more stimulus features could potentially modulate the behavioral response, sharpening or reducing it, and it is not restricted to vision only.

As a possible alternative to this current SNN model, the accumulation of sensory inputs across spatio-temporal changes in the movement of the visual stimulus could boost visual signal. In that sense, a vertical stimulus that moves from left to right direction scans a larger portion of the retina instead of an horizontal stimulus. If more sensory neurons are triggered, these extra inputs may also enhance or sharpen the response of the direction cells.

In this experiment, the robot displayed a faster motor output from simultaneously integrating the orientation and direction information of a visual stimulus. Other form of behavioral enhancements could certainly be drawn. For example, benefits could be anticipated from better accuracy, stronger intensity or a faster motor response of organisms; from a barely noticeable gain to a major survivability impact. As such, this paper represents a first step model tested in a static robotic context, but more realistic and dynamical scenarios still remain to be studied. Thus, the present study is limited in terms of motor behavioral complexity, though we believe that the core of the SNN would not change by any addition in the output, since the supervised reward is based on any manifestation of an appropriate response. Also, using visual stimuli with a dynamical robot often requires scaling and focus strategies that were beyond the scope of this article, but may be considered in future work.

Currently, the proposed SNN model contains highly designed connections which reflects the complexity and diversity of biological models (Briggman et al., 2011; Masland, 2012; Kim et al., 2014; Wernet et al., 2014; Demb and Singer, 2015; Fitzgerald and Clark, 2015; Ding et al., 2016; Serbe et al., 2016; Vlasits et al., 2016), but other computational SNN model could be elaborated to obtain more similarity of biological models. In this perspective, instead of using built-in synaptic connections that respond to pure black or white tones, artificial ganglion's cells could be integrated to mimic on-off-center receptive fields and dark/light edges motion (Joesch et al., 2010; Borst and Euler, 2011; Meier et al., 2014; Takemura et al., 2017). Another bio-inspired approach to integrate orientation could be to introduce a suppressive mechanism using inhibitory connections to enhance the direction neural response. Thus, comparative experiments between the biological, computational and robotic model still need to be explored.

In the present SNN model, only two different black bar orientations were used to simplify the process. Also, the simulations were done with a defined constant speed of moving stimuli. Expanding the current model to cope with all orientations is a matter of scaling units and synapses, but would not alter outcome since only two sensory neurons on two axis are needed to obtain the orientation feature. Affording all motion dynamics of stimuli (Li et al., 2017) is perhaps more complex. This remains to be studied, given that in the MD model, the SNN computational method and the STDP learning rules are intrinsically sensible to temporal aspects. In this perspective, variation in the timing of the reinforcement and its schedule as well as extending the STDP period limitation remain to be studied. Another interesting alternative would be to use the same amount of units present in the current architecture, but allow them to respond with a differential firing rate to changes in stimulus orientation, similarly observed in biological networks.

## 5. Conclusion

Following the recent evidence in vision neuroscience, this work focused on the effect of merging visual orientation and direction processes in a MD computational robotic model. The model was simulated with an SNN method and implemented in a robotic learning context to validate the results at the behavioral level. Specifically, the SNN learned the association between a particular action and a motion visual stimulus from rewards. Both the virtual and physical world experiments succeeded in showing an acceleration of the motor response onset when the visual stimulus orientation is orthogonal to its motion.

## Author contributions

AC did the most of review of literature. AC and FT elaborated the design of the study, the neural architecture and made the virtual simulation. They did most of the data analysis and article redaction. MR contributed in the physical simulation and he reviewed the article as well as providing help in the development of the neural architecture. NB and SC critically enhanced the article (in its structure and with ideas and directions).

### Conflict of interest statement

The authors declare that the research was conducted in the absence of any commercial or financial relationships that could be construed as a potential conflict of interest.

## Supplementary material

Table S1 Neural network parameter values.

Table S2 Synaptic parameter values.
